# Host Immunity to Mycobacterium tuberculosis Infection Is Similar in Simian Immunodeficiency Virus (SIV)-Infected, Antiretroviral Therapy-Treated and SIV-Naïve Juvenile Macaques

**DOI:** 10.1128/iai.00558-22

**Published:** 2023-04-11

**Authors:** Erica C. Larson, Amy L. Ellis, Mark A. Rodgers, Abigail K. Gubernat, Janelle L. Gleim, Ryan V. Moriarty, Alexis J. Balgeman, Yonne K. Menezes, Cassaundra L. Ameel, Daniel J. Fillmore, Skyler M. Pergalske, Jennifer A. Juno, Pauline Maiello, Alexander G. White, H. Jacob Borish, Dale I. Godfrey, Stephen J. Kent, Lishomwa C. Ndhlovu, Shelby L. O’Connor, Charles A. Scanga

**Affiliations:** a Department of Microbiology and Molecular Genetics, University of Pittsburgh School of Medicine, Pittsburgh, Pennsylvania, USA; b Center for Vaccine Research, University of Pittsburgh School of Medicine, Pittsburgh, Pennsylvania, USA; c Department of Pathology and Laboratory Medicine, University of Wisconsin - Madison, Wisconsin, USA; d Department of Immunobiology, Federal University of Santa Catarina, Florianópolis, Santa Catarina, Brazil; e Department of Microbiology and Immunology, The Peter Doherty Institute for Infection and Immunity, University of Melbourne, Melbourne, Victoria, Australia; f Melbourne Sexual Health Centre and Department of Infectious Diseases, Alfred Hospital and Centre Clinical School, Monash University, Melbourne, Victoria, Australia; g Department of Medicine, Division of Infectious Disease, Weill Cornell Medicine, New York, New York, USA; h Wisconsin National Primate Research Center, University of Wisconsin - Madison, Wisconsin, USA; Weill Cornell Medicine

**Keywords:** HIV/TB coinfection, HIV, TB, T cells, antiretroviral therapy (ART), pediatrics, pediatric immunology

## Abstract

Pre-existing HIV infection increases tuberculosis (TB) risk in children. Antiretroviral therapy (ART) reduces, but does not abolish, this risk in children with HIV. The immunologic mechanisms involved in TB progression in both HIV-naive and HIV-infected children have not been explored. Much of our current understanding is based on human studies in adults and adult animal models. In this study, we sought to model childhood HIV/Mycobacterium tuberculosis (Mtb) coinfection in the setting of ART and characterize T cells during TB progression. Macaques equivalent to 4 to 8 year-old children were intravenously infected with SIVmac239M, treated with ART 3 months later, and coinfected with Mtb 3 months after initiating ART. SIV-naive macaques were similarly infected with Mtb alone. TB pathology and total Mtb burden did not differ between SIV-infected, ART-treated and SIV-naive macaques, although lung Mtb burden was lower in SIV-infected, ART-treated macaques. No major differences in frequencies of CD4^+^ and CD8^+^ T cells and unconventional T cell subsets (Vγ9+ γδ T cells, MAIT cells, and NKT cells) in airways were observed between SIV-infected, ART-treated and SIV-naive macaques over the course of Mtb infection, with the exception of CCR5+ CD4^+^ and CD8^+^ T cells which were slightly lower. CD4^+^ and CD8^+^ T cell frequencies did not differ in the lung granulomas. Immune checkpoint marker levels were similar, although ki-67 levels in CD8^+^ T cells were elevated. Thus, ART treatment of juvenile macaques, 3 months after SIV infection, resulted in similar progression of Mtb and T cell responses compared to Mtb in SIV-naive macaques.

## INTRODUCTION

Pediatric tuberculosis (TB) caused by the bacterium, Mycobacterium tuberculosis (Mtb), is a major global health concern. In 2019, around 1.2 million children under the age of 15 fell ill with TB and over 200,000 children died of TB, including children with HIV-associated TB (1). HIV-infected children have higher rates of mortality due to TB than HIV-uninfected children (2). Children account for roughly 10% of HIV-associated TB deaths, which amounted to ~20,000 lives in 2020 (1, 2). Antiretroviral therapy (ART) reduces TB risk and mortality by suppressing viral replication and restoring CD4^+^ T cell levels, but TB risk does not completely return to the level seen in HIV-naive children (3–7). Moreover, pediatric TB often manifests differently than adults and disease progression is influenced by age (8, 9). Miliary TB and TB meningitis is more common in infants and young children (<2 years old), while pulmonary TB is more common in older children (9). HIV infection exacerbates TB disease in children and is associated with greater lung involvement and cavitation regardless of age (10, 11). Given the severity of TB in children, especially those with HIV, there is a clear need to elucidate immune mechanisms underlying TB progression in children as it may help inform diagnostic and treatment strategies.

Much of what is known about pediatric TB is through the lens of human adult studies and adult animal models. However, this overlooks the dynamic nature of the developing, pediatric immune system (12). Throughout childhood, T cell composition is incredibly dynamic and does not stabilize until adulthood (13–16). Rapid accumulation of circulating memory CD4^+^ and CD8^+^ T cells occurs during the first few years of life in both children and young nonhuman primates (NHP) (13, 17, 18). Given that CD4^+^ and CD8^+^ T cells are critical for Mtb control (19, 20) and the predominant immature nature of CD4^+^ and CD8^+^ T cells during the first few years of life, this may be a contributing factor to severe TB disease observed in young children. However, the role of CD4^+^ and CD8^+^ T cells in TB pathogenesis in children is largely understudied. In addition, HIV infection is well-known to cause CD4^+^ and CD8^+^ T cell dysfunction through CD4^+^ T cell depletion and T cell exhaustion (21–24). ART has been shown to restore CD4^+^ T cell levels and improve CD8^+^ T cell function, but the immune restoration is incomplete (25–28). Similarly, unconventional T cells, such as γδ T cells, MR1-restricted mucosal-associated invariant T (MAIT) cells, and CD1d-restricted natural killer T (NKT) cells have received little attention in pediatric TB despite their ability to recognize non-peptide Mtb antigens and may play a possible role in early Mtb control (29–33). Vδ2+ γδ T cells, a subset of γδ T cells which forms T cell receptor heterodimers with Vγ9, and MAITs are virtually absent in early life in humans (16). Moreover, unconventional T cell subsets are depleted during HIV infection and only partially restored by ART (34–36). Whether their role in TB progression differs between HIV, ART-treated children and HIV-naive children has yet to be thoroughly investigated.

NHP are an excellent model to study TB as they closely recapitulate the immune responses and pathogenesis observed in humans (37, 38). NHP are also susceptible to SIV, a close relative of HIV, which results in HIV-like disease progression and AIDS development in some macaque species (39). Previously, in adult Mauritian cynomolgus macaques, we found that Mtb coinfection of ART-naive, SIV-infected animals had worsened TB disease compared to SIV-naive macaques (40), in alignment with studies in humans (41–43). In a separate study, we observed granulomas obtained from SIV/Mtb coinfected macaques early in the course of Mtb infection had immunologic differences compared to animals infected with Mtb alone, such as fewer CD4^+^ T cells, more CD8^+^ T cells, and elevated frequencies of PD-1+ and TIGIT+ T cells, indicative of chronic immune activation (44). Although these studies inform our understanding of TB immunity in coinfected adults, very few NHP studies to date have modeled pediatric TB (45–47).

This is the first study to characterize CD4^+^ and CD8^+^ T cell populations over the course of infection using an NHP model of pediatric TB and HIV/Mtb coinfection. Juvenile macaques, ~ 1 to 2 years of age (equivalent to 4 to 8 years in humans), were either infected with Mtb alone or were infected with SIV, treated with ART, and then coinfected with Mtb. We found very few differences in TB disease progression and Mtb burden between SIV-infected, ART-treated and SIV-naive macaques. While we did observe immunological changes following SIV infection, such as fewer CD4^+^ T cells and more CD8^+^ T cells in airways, these returned to pre-SIV levels following ART initiation. The frequencies of CD4^+^ and CD8^+^ T cells in airways remained similar between SIV-infected, ART-treated and SIV-naive macaques 8 weeks after Mtb infection. Frequencies of unconventional T cell subsets (Vγ9+ γδ T cells, MAIT cells, and NKT cells) did not differ over the course of SIV nor between SIV-infected, ART-treated and SIV-naive macaques following Mtb infection. Similarly, T cell composition of granulomas did not differ between the 2 groups. Thus, juvenile macaques treated with ART within 3 months of SIV infection appear to experience similar TB progression and mount similar T cell responses to Mtb coinfection as animals infected with Mtb alone.

## RESULTS

### T cell subsets and phenotype of CD4+and CD8^+^ T cells in airways do not differ, except for CCR5, between SIV-infected, ART-treated, and SIV-naive juvenile macaques.

**FIG 1:**
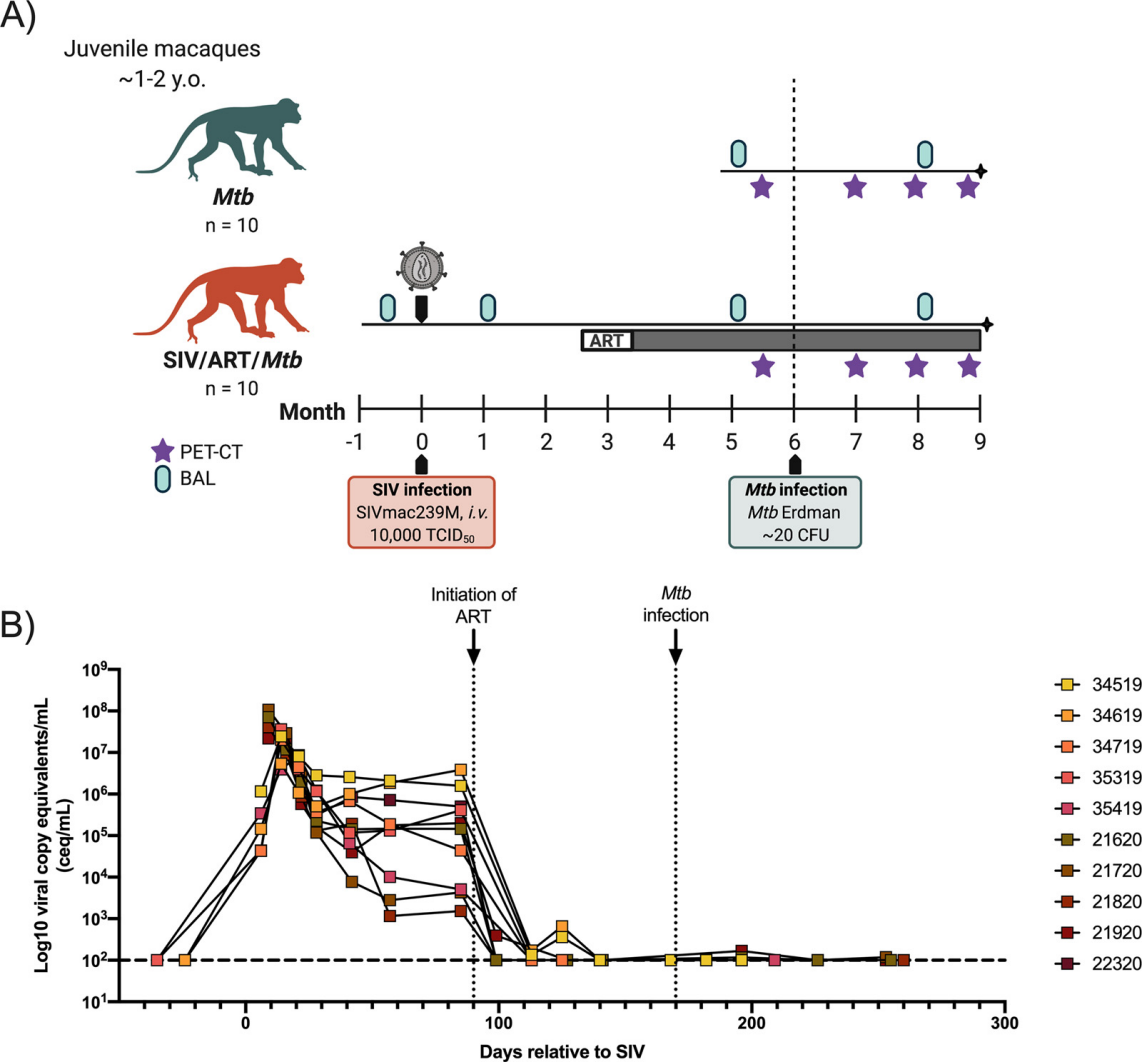
Timeline of juvenile SIV-infected, ART-treated Mtb co-infection study and plasma viremia of SIV-infected, ART-treated macaques over the course of SIV and Mtb coinfection. (A) Study timeline. (B) Plasma viral copy equivalents were determined by qRT-PCR. Each point indicates an individual animal. Horizontal dashed line represents the limit of detection.

Ten juvenile macaques were infected intravenously with SIVmac239M (Fig. 1A) and, as expected, peak viremia was detected approximately 10 days postinfection, followed by viral load reduction and establishment of set-point viremia, which varied widely among animals. Three months after infection, ART was initiated in all animals and reduced viremia to below the limit of detection (Fig. 1B). Plasma viral load remained undetectable in all animals after ART initiation, with the exceptions of 34519 and 34619 which had transient viremia that did not exceed 10^3^ viral copies (Fig. 1B). All 10 SIV-infected, ART-treated animals, as well as 10 age-matched SIV-naive animals, were then infected with approximately 5 to 11 CFU of a barcoded Mtb (Table S1). ART continued to suppress viral replication after Mtb challenge in SIV-infected animals (Fig. 1B).

**FIG 2:**
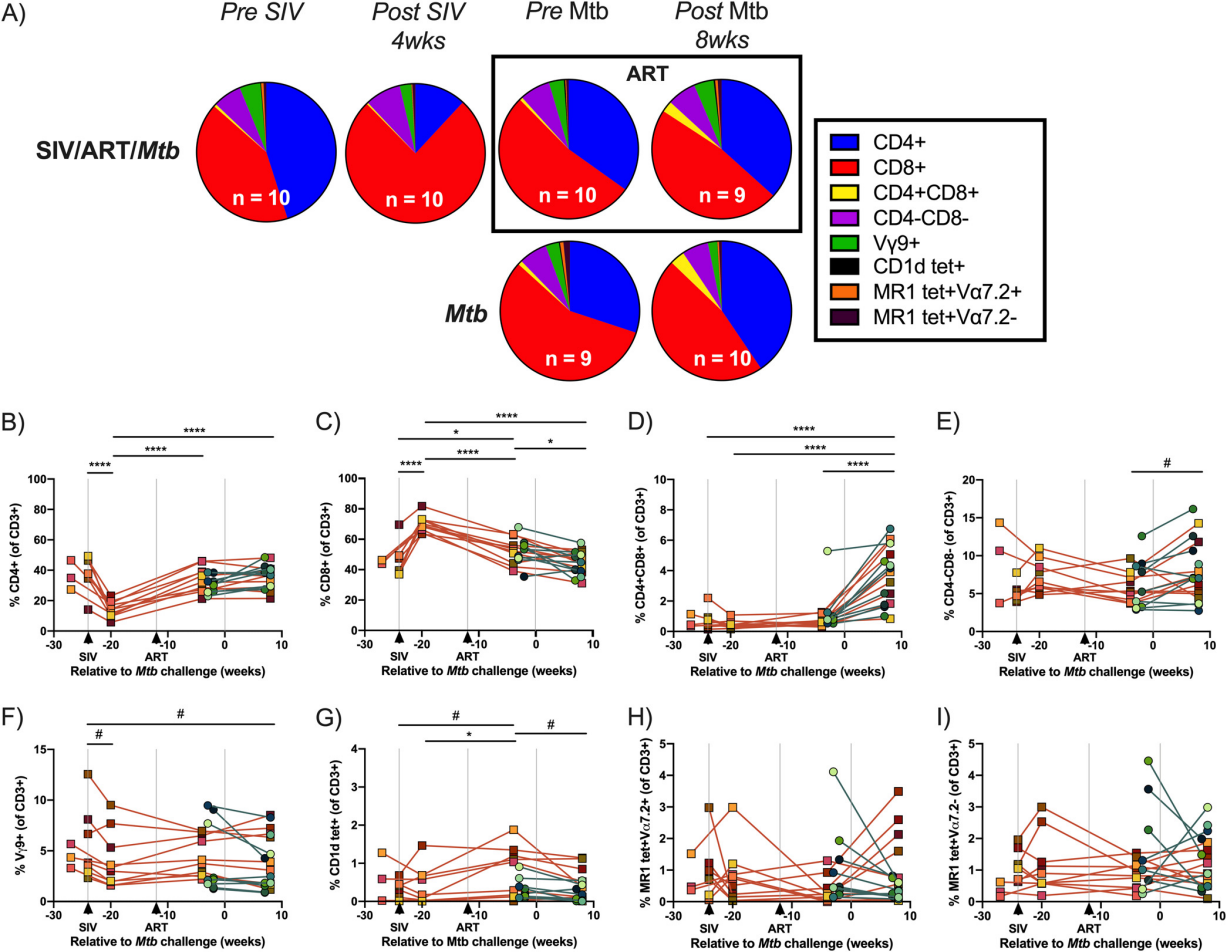
Composition of T cell subsets in airways over time. BALs were collected over the course of SIV and Mtb infection. (A) Proportions of T cell subsets by median event counts. BALs collected after ART are outlined in black. (B) to (I) Frequencies of T cell subsets over time relative to CD3^+^ gate. Individual animals indicated by symbols. SIV-infected, ART-treated Mtb coinfected macaques are indicated by orange lines and macaques infected with Mtb alone are indicated by teal lines. A mixed statistical model was performed to determine significance with fixed effects (time and group) and random effects (individual animals). Bars with asterisks indicate significance between time points; #, 0.05 < *P* < 0.1; *, *P* < 0.05; and ****; *P* < 0.0001. Fixed effect test results and Tukey pairwise comparisons are included in Table S4.

Mtb is predominantly transmitted through inhaled droplets and first encounters host immune cells when deposited in the airways. Thus, we assessed T cell composition and characterized T cell phenotype in the airways of the animals during SIV infection and ART treatment by flow cytometric analysis of cells recovered by bronchoalveolar lavage (BAL). The composition of T cell subsets appeared to dramatically shift following SIV infection, with a notable decrease in the CD4^+^ proportion (blue) and a corresponding increase in the CD8^+^ proportion (red) (Fig. 2A, top row). The T cell composition shifted again following ART, returning to proportions similar to those observed prior to SIV infection (Fig. 2A). T cell proportions were similar between SIV-infected, ART-treated macaques (Fig. 2A, top row) and macaques infected with Mtb alone (Fig. 2A, bottom row), both prior to and 8 weeks after Mtb infection (Fig. 2A). It should be noted that Fig. 2A is a visual representation of the distribution of cell types and no formal statistics were performed. However, these changes in T cell distribution were noted when data are presented as frequencies of the total CD3^+^ population (Fig. 2B to J). We observed a significant decline in CD4^+^ T cells with a concomitant rise in CD8^+^ T cell frequencies in the airways following SIV infection and a return to pre-SIV frequencies after initiating ART (Fig. 2B and C). At the time of Mtb coinfection, CD4^+^ T cell frequencies in BAL were similar between SIV-infected, ART-treated animals and SIV-naive controls and did not change appreciably after Mtb infection (Fig. 2B). CD8^+^ T cells exhibited a subtle, but significant, decline in both groups following Mtb coinfection (Fig. 2C). CD4+CD8^+^ T cell frequencies increased following Mtb infection in both groups (Fig. 2D), which has been reported previously (48). We did not observe significant differences in CD3+CD4-CD8- T cells or consistent changes in unconventional T cell subsets, including γδ T cells (Vγ9+), NKT cells (CD1d tetramer+), MAIT cells (MR1 tetramer+ Vα7.2+), and MAIT-like cells (MR1 tetramer+ Vα7.2-) between the 2 groups following Mtb coinfection (Fig. 2E to I).

We did observe some phenotypic changes in CD4^+^ and CD8^+^ T cells in airways over the course of the study. There was a transient spike in proliferation, as measured by ki-67, in CD4^+^ and CD8^+^ T cells at 4 weeks post SIV infection which then subsided following ART to levels similar to those observed in uninfected macaques (Fig. S1A and B). CD4^+^ and CD8^+^ T cell proliferation was previously shown to correspond to the rise in viral replication during acute SIV infection (49–51). Following Mtb infection, the frequency of PD-1+ CD4^+^ T cells, but not PD-1+ CD8^+^ T cells, declined significantly (Fig. S1C and D). The frequency of TIGIT+ CD4^+^ and CD8^+^ T cells fluctuated, with notable increases in frequency of TIGIT+ CD4^+^ T cells 4 weeks post SIV infection and of TIGIT+ CD8^+^ T cells following Mtb infection (Fig. S1E and F). A small, but significant, drop in the frequency of CXCR3+ CD8^+^ T cells, but not CD4^+^ T cells, was noted following Mtb coinfection (Fig. S1G and H). CCR6-expressing CD4^+^ and CD8^+^ T cells significantly increased in frequency in both groups following Mtb coinfection (Fig. S1I and J). As CCR6 mediates cell migration during inflammation and immune response (52), this increased frequency of CCR6+ CD4^+^ and CD8^+^ T cells most likely indicates enhanced trafficking to the airways in response to Mtb (53).

**FIG 3:**
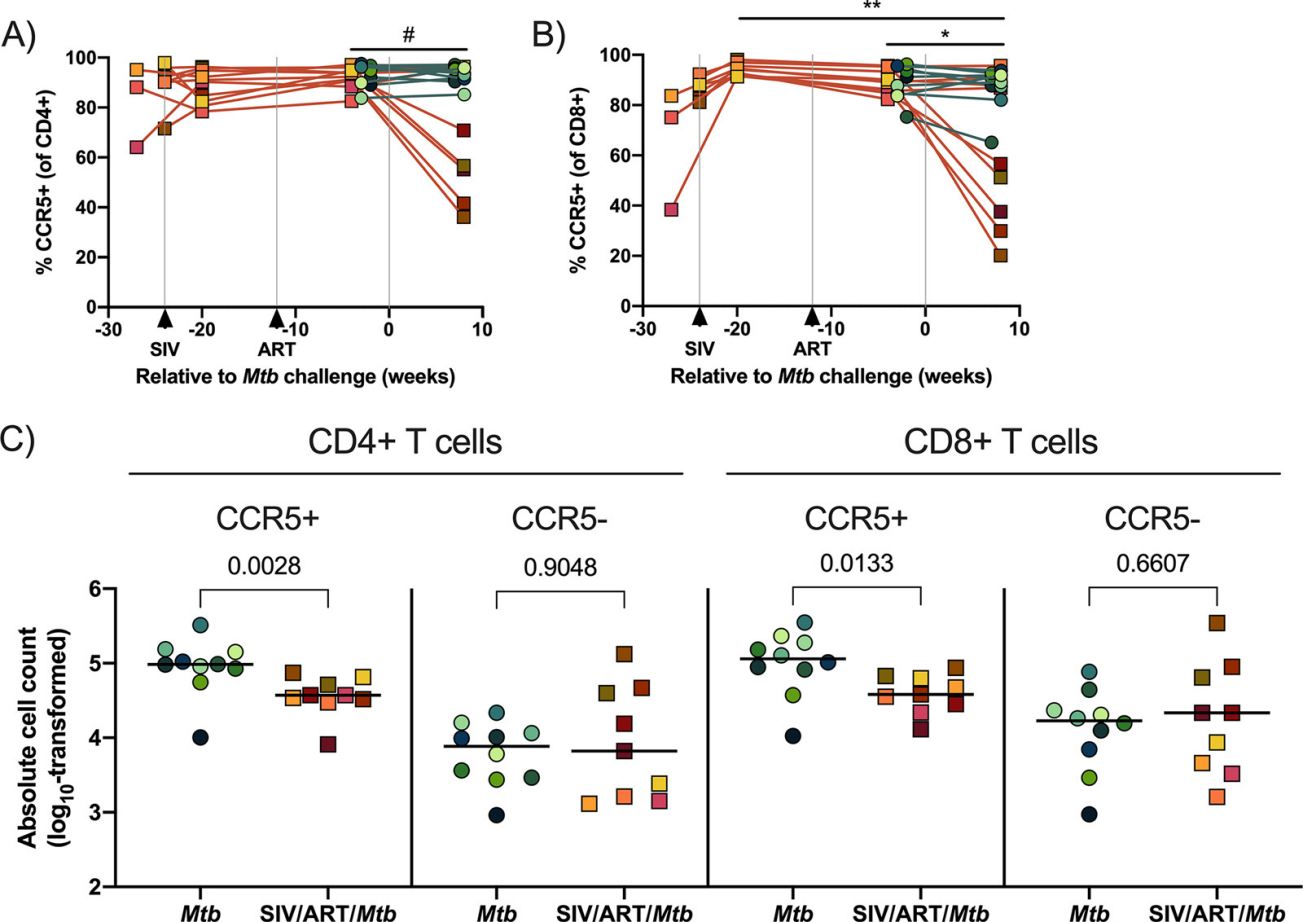
CCR5+ CD4^+^ and CD8^+^ T cells in airways at 8 weeks post Mtb infection. BAL cells were collected over the course of SIV and at 8 weeks post Mtb infection and stained for flow cytometry. (A) and (B) Individual animals indicated by symbols. SIV-infected, ART-treated Mtb coinfected macaques are indicated by orange lines and macaques infected with Mtb alone are indicated by teal lines. A mixed statistical model was performed to determine significance with fixed effects (time and group) and random effects (individual animals). Bars with asterisks indicate significance between time points; #, 0.05 < *P* < 0.1; *, *P* < 0.05; and **, *P* < 0.01. Fixed effect test results and Tukey pairwise comparisons are included in Table S4. (A) Frequencies of CCR5+ CD4^+^ T cells over the course of the study. (B) Frequencies of CCR5+ CD8^+^ T cells over the course of the study. (C) Absolute CCR5+/− CD4^+^ and CD8^+^ T cells in airways at 8 weeks post Mtb. Absolute cell counts were calculated from the hemacytometer cell count. Individual samples indicate individual animals and bars indicate group medians. Mann-Whitney U tests was performed to determine significance between groups. *P* values are shown.

The frequency of CCR5, on the other hand, significantly declined in CD8^+^ T cells, and to a lesser extent in CD4^+^ T cells (*P* = 0.0534), in SIV-infected, ART-treated macaques following Mtb coinfection (Fig. 3A and B). CCR5 is a coreceptor utilized by HIV/SIV for infection of CD4^+^ T cells and is closely linked to T cell loss (54, 55). This decline was also reflected by a loss of absolute CCR5+ CD4^+^ and CD8^+^ T cell numbers in the airways (Fig. 3C). SIV-infected, ART-treated animals had significantly fewer CD4^+^ and CD8^+^ T cells in their airways (~ 0.39 and 0.40 log decrease, respectively) than compared SIV naive animals (Fig. 3C). Given the role of CD4^+^ and CD8^+^ T cells in Mtb control, we were interested in whether this subtle loss of conventional T cells in the airways impacted overall TB progression.

### No difference in lung inflammation or Mtb dissemination.

**FIG 4:**
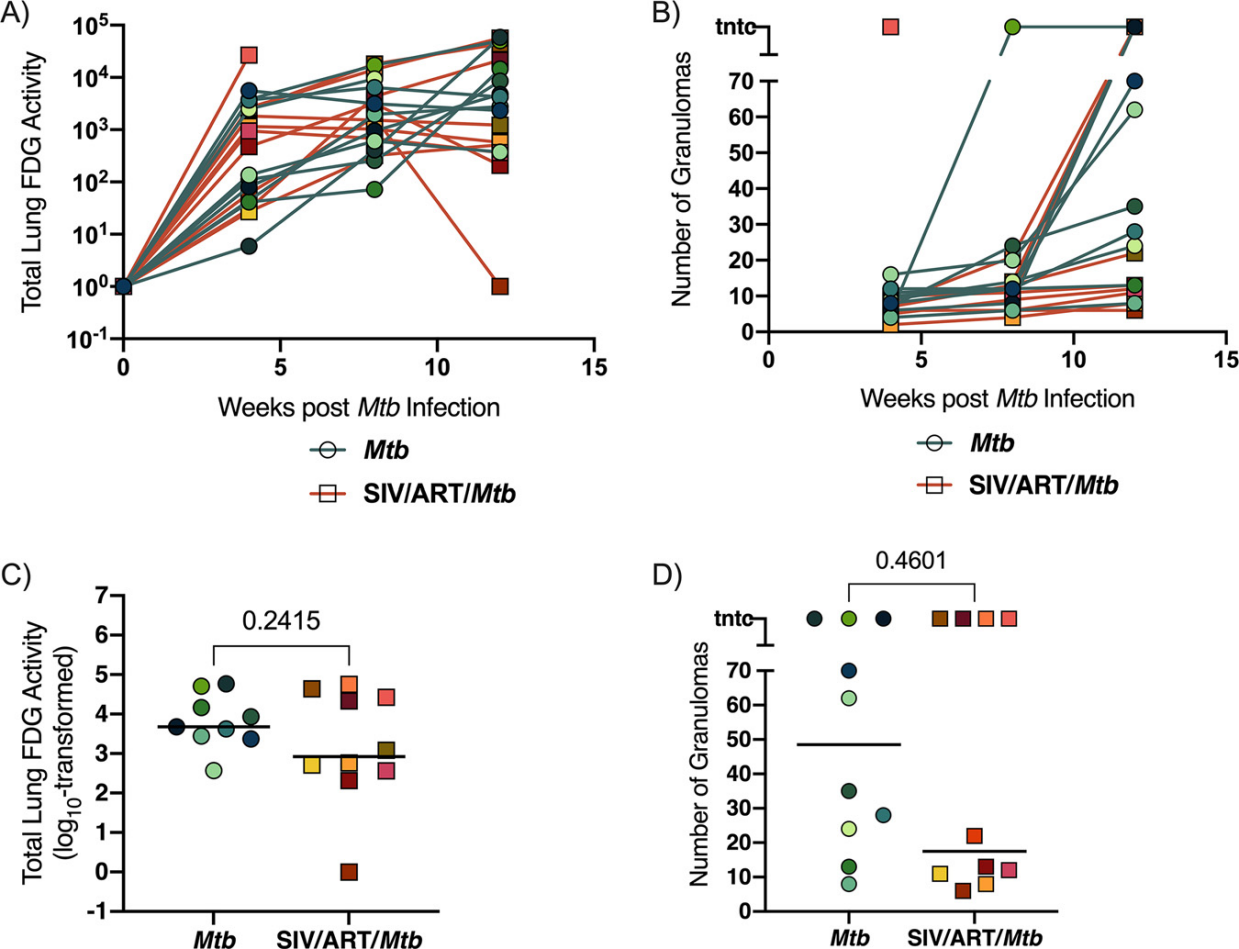
TB progression by PET/CT imaging. (A) Lung FDG activity over time. SIV-infected, ART-treated Mtb coinfected macaques (orange lines) and macaques infected Mtb alone (teal lines). Individual animals are shown. (B) The number of granulomas identified by PET-CT at 4, 8, and 12 weeks (pre-necropsy). SIV-infected, ART-treated Mtb coinfected macaques (orange lines) and macaques infected Mtb alone (teal lines). Individual animals are shown. (C) Total lung FDG activity at time of necropsy. Bars indicate medians of group. An unpaired t test was performed to compare groups. *P* value is shown. (D) The number of granulomas identified at necropsy (12 wks *p.i. Mtb*). Bars indicate medians of group. A Mann-Whitney U test was performed. *P* value is shown.

We previously reported a dramatic increase in lung inflammation and Mtb dissemination in SIV-infected, ART-naive adult macaques between 4 and 8 weeks post Mtb compared to adult macaques infected with Mtb alone, indicating a loss of Mtb control in the SIV-infected group (40). Here, we used PET/CT to determine whether lung inflammation and Mtb dissemination differed over the course of Mtb infection in our juvenile SIV-infected, ART-treated macaques compared to juvenile macaques infected with Mtb alone. We measured FDG uptake, a surrogate for inflammation, to quantify lung inflammation and to enumerate granulomas over time as a measure of Mtb dissemination (Fig. 4). SIV-infected, ART-treated macaques coinfected with Mtb did not differ from macaques infected with Mtb alone in terms of total lung FDG activity over the course of Mtb infection (Fig. 4A). Similarly, both groups exhibited similar numbers of granulomas over the Mtb infection course (Fig. 4B). One SIV-infected, ART-treated animal had rapidly progressive TB disease and reached humane endpoint just 6 weeks post Mtb coinfection and is represented as a single datapoint at 4 weeks. When measured at the final time point, there were no significant differences in lung inflammation (*P* = 0.2415) or the number of granulomas (*P* = 0.4601) between SIV-infected, ART-treated macaques coinfected with Mtb, and macaques infected with Mtb alone (Fig. 4C and D), indicating similar TB progression in the 2 groups.

### TB pathology, bacterial burden, and bacterial dissemination were similar in SIV-infected, ART-treated, and SIV-naive juvenile macaques, except for lung CFU.

**FIG 5:**
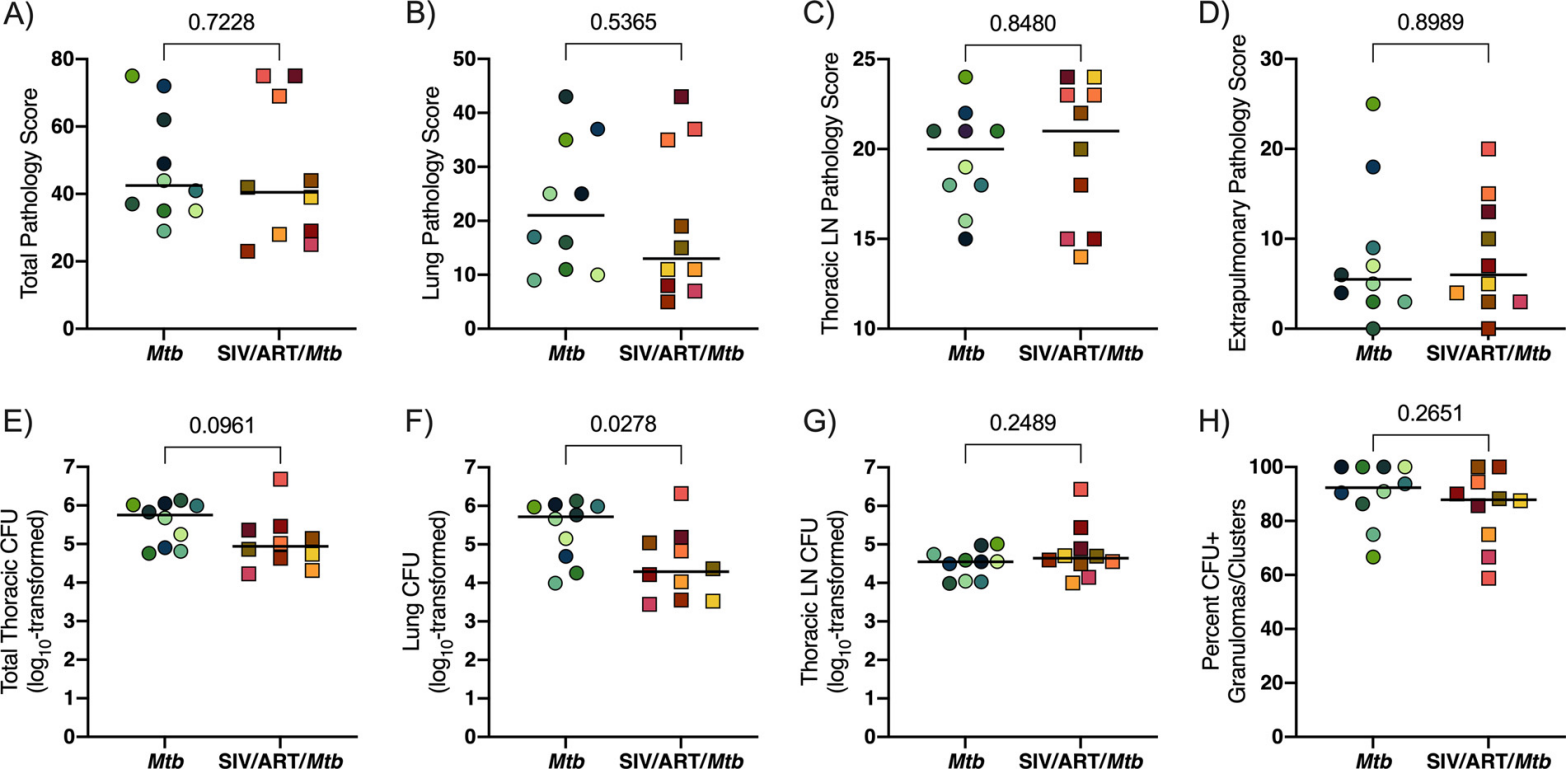
TB pathology and CFU at 12 weeks post Mtb infection. Bars indicate medians of group. Unpaired t tests of group medians were performed. *P* values are shown. (A) Total pathology score. (B) Lung pathology score. (C) Thoracic lymph node pathology score. (D) Extrapulmonary pathology score. (E) Total thoracic CFU (lung CFU + thoracic lymph nodes CFU). (F) Lung CFU. (G) Thoracic lymph nodes CFU. (H) Percent CFU+ granulomas or clusters.

Following Mtb infection, erythrocyte sedimentation rates were normal in both SIV-infected, ART-treated macaques, and SIV-naive macaques while culturable bacilli were variably detected in BAL and gastric aspirates from both groups (Table S1). At necropsy, we used an established scoring system to assess total TB pathology across several tissue compartments: lungs, thoracic lymph nodes, and extrapulmonary sites (56). While the individual pathology scores varied widely, there were no significant differences in the group medians between SIV-infected, ART-treated macaques coinfected with Mtb and macaques infected with Mtb alone (Fig. 5A to D).

We plated tissue samples for CFU to determine Mtb burden. Somewhat surprisingly, the median total thoracic burden of Mtb, comprised of CFU from both lung and thoracic lymph nodes, was slightly lower in SIV-infected, ART-treated macaques compared to macaques infected with Mtb alone although this difference did not reach statistical significance (*P* = 0.0961) (Fig. 5E). Interesting, the bacterial load was significantly lower when only the lungs were considered (*P* = 0.0278) (Fig. 5F). In contrast, the median bacterial load in thoracic lymph nodes between the groups was similar (*P* = 0.2489) (Fig. 5G). Culture-negative lung granulomas identified at necropsy were considered to have been sterilized by the host. Both groups had similar percentages of lung granulomas with culturable bacilli, indicating comparable capacity to eliminate viable Mtb in SIV-infected, ART-treated animals and those that were infected with Mtb alone (Fig. 5H).

To assess Mtb dissemination, Mtb DNA was isolated from CFU+ tissue samples and the number and distribution of uniquely tagged bacilli was quantified across individual animals and tissue types. We did not find differences in the median number of uniquely tagged bacilli per animal between cohorts (Fig. S2A). The number of uniquely tagged bacilli identified in granulomas and thoracic lymph nodes did not differ between SIV-naive and SIV-infected, ART-treated animals (Fig. S2B). However, consistent with previous reports of lymph node seeding from multiple granulomas (57, 58), we observed a significantly higher number of uniquely tagged bacilli in thoracic lymph nodes when compared to granulomas. This was the case for both SIV-naive (*P* = 0.0397) and SIV-infected, ART-treated animals (*P* = 0.0002) indicating similar dissemination between these groups.

**FIG 6:**
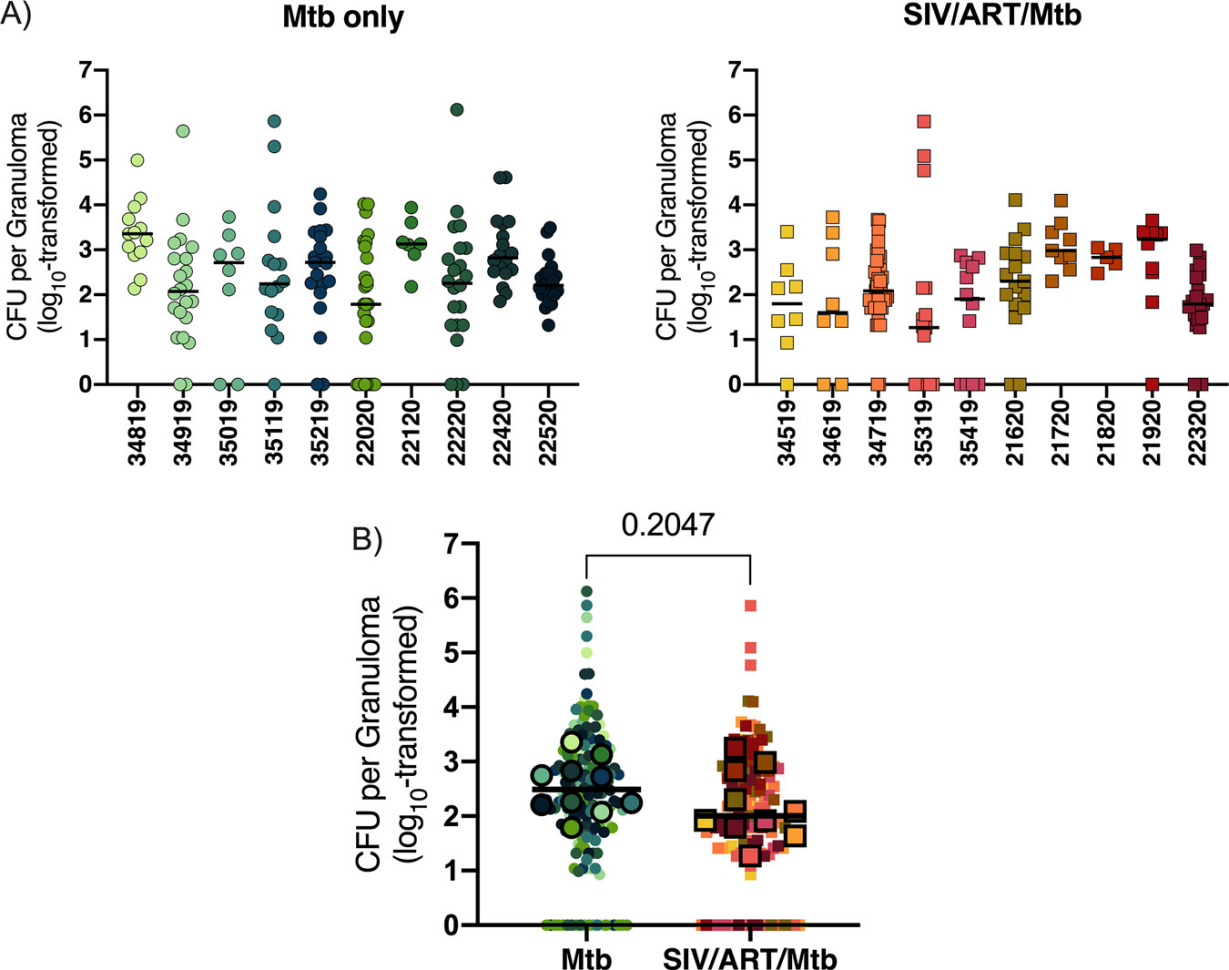
Bacterial burden in individual granulomas. (A) CFU per granuloma by animal. Symbols represent CFU from individual granulomas. Bars indicate median CFU per animal. Mtb only animals (A, left panel) and SIV/ART/Mtb animals (A, right panel). (B) Combined CFU for Mtb only and SIV/ART/Mtb groups. Outlined symbols indicate median per animal and non-outlined symbols indicate individual granulomas. Bars indicate median CFU per animal per group and an unpaired t test was performed to determine significance. The *P* value is shown.

We also analyzed the bacterial load in individual granulomas from each animal. The number of culturable Mtb within individual granulomas varied widely both within individual animals, as well as between animals from each group (Fig. 6A). However, there was no significant difference in the median CFU of individual granulomas between those from SIV-infected, ART-treated macaques coinfected with Mtb, and those from macaques infected with Mtb alone (*P* = 0.2047; Fig. 6B). Thus, both the severity of TB, Mtb bacterial load, and Mtb dissemination were similar in SIV-naive and SIV-infected, ART-treated animals.

### Cytokine responses to Mtb-specific antigens were similar in the lungs of SIV-infected, ART-treated macaques coinfected with Mtb and macaques infected with Mtb alone.

**FIG 7:**
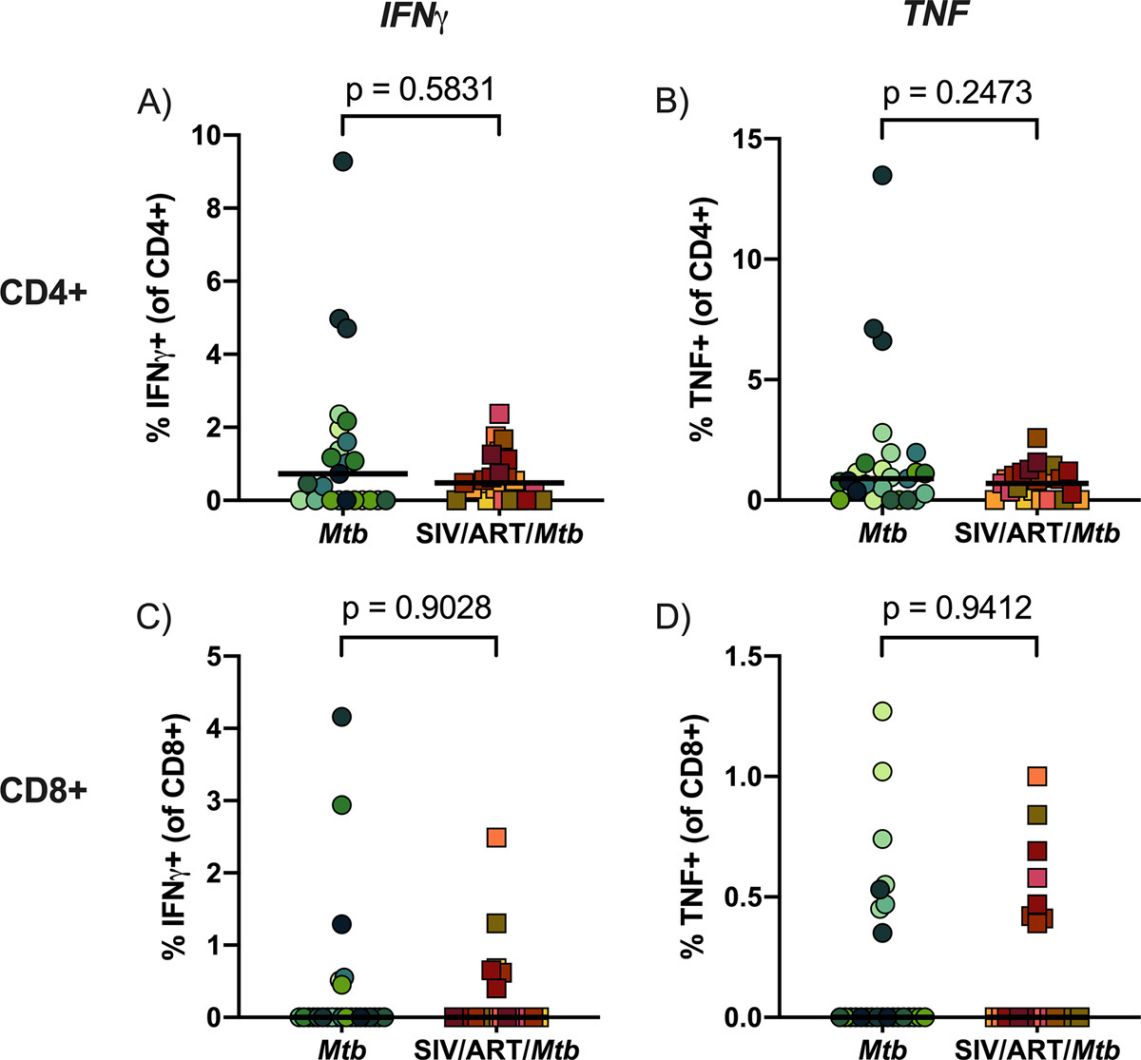
Cytokine responses in lung tissue. Lung tissue was stimulated with H37Rv whole cell lysate for 14 h. Cytokine production was corrected for against unstimulated controls. Bars indicate medians of pooled data. Mann-Whitney U tests were performed to determine significance. *P* values are shown. (A) IFNγ production in CD4^+^ T cells. (B) TNF production in CD4^+^ T cells. (C) IFNγ production in CD8^+^ T cells. (D) TNF production in CD8^+^ T cells.

Cell suspensions were prepared from lung tissue without apparent granulomas irrespective of whether the tissue would be determined to have culturable Mtb. The cells were stimulated with Mtb whole cell lysate to assess CD4^+^ and CD8^+^ T cell responses to Mtb-specific antigens in the lung (Fig. 7). There were no differences in IFNγ or TNF production between SIV-infected, ART-treated macaques coinfected with Mtb and macaques infected with Mtb alone in either CD4^+^ and CD8^+^ T cells (Fig. 7A to D), indicating similar Mtb-specific cytokine responses between the 2 groups.

### Granuloma composition and phenotype did not differ between Mtb-infected SIV-infected, ART-treated, and SIV-naive macaques.

Granulomas are the hallmark of TB disease. While normal granuloma formation is known to be affected in HIV/Mtb coinfected adults (59, 60), very little is known about TB granuloma formation dynamics and composition in children coinfected with HIV, regardless of whether they are treated with ART or not. Histopathology of excised granulomas was assessed by an experienced veterinary pathologist and no generalizable histological differences could be determined in the granulomas from SIV-naive and SIV-infected, ART-treated animals (data not shown).

**FIG 8:**
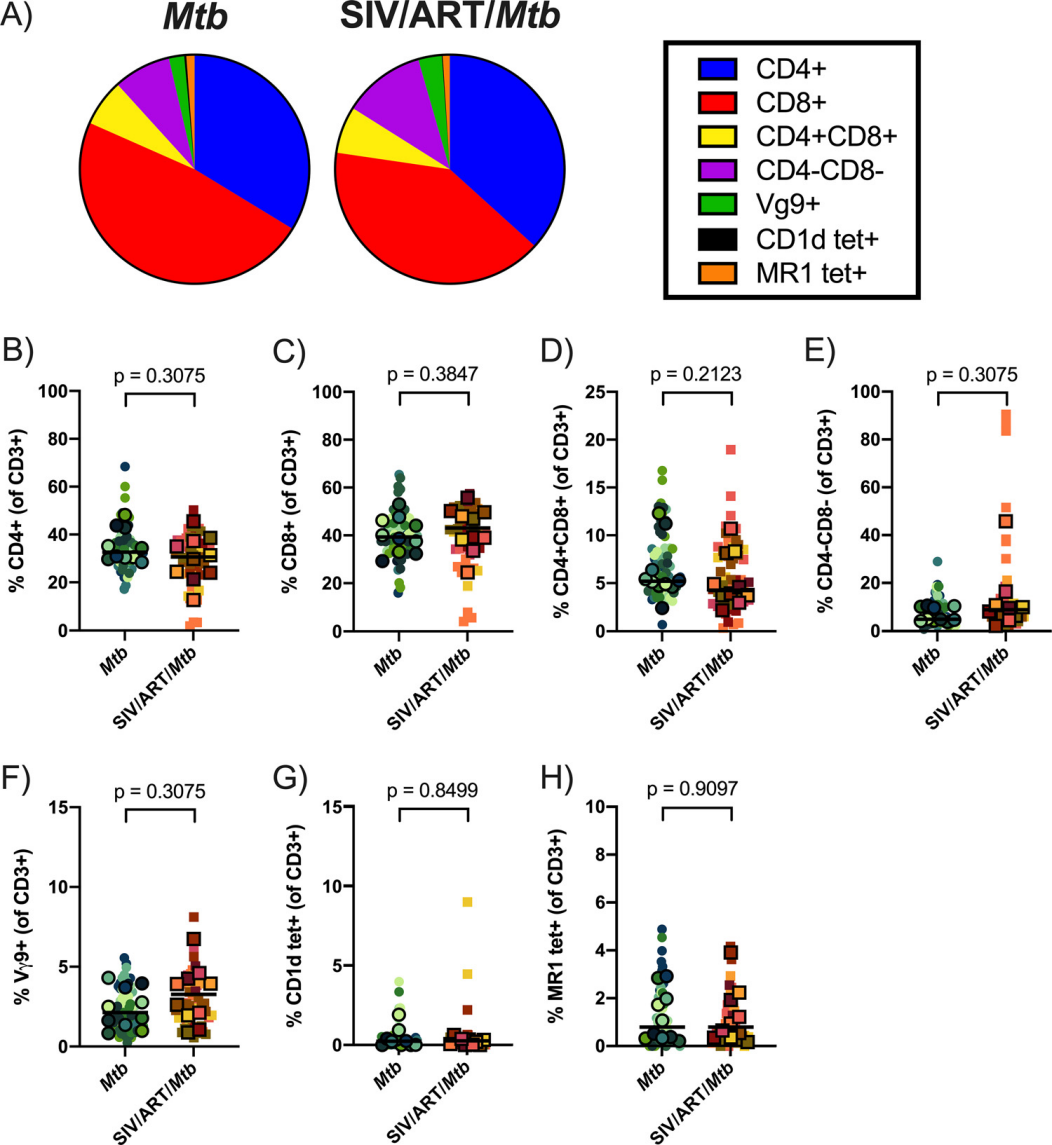
T cell subsets in granulomas. (A) Proportion of T cell subsets in individual granulomas by median event counts. (B) to (H) Frequencies of T cell subsets in granulomas relative to CD3^+^ gate. (B) CD4^+^ T cells. (C) CD8^+^ T cells. (D) CD4+CD8^+^ T cells. (E) CD4-CD8- T cells. (F) Vγ9+ T cells. (G) CD1d tet+ T cells. (H) MR1 tet+ T cells. Outlined symbols indicate median per animal and unlined symbols indicate individual samples. Bars indicate group medians. Wilcoxon tests of group medians were performed to determine significance. *P* values are shown.

We used flow cytometry to compare the cellular composition of TB granulomas in lungs of SIV-infected, ART-treated macaques with those in SIV-naive macaques. We did not observe any difference in overall T cell composition (Fig. 8A) or in frequencies of T cell subsets (Fig. 8B to H). While most T cell subset frequencies did not differ in other tissue compartments (lung, lymph nodes, spleen, and blood), we observed a slight decrease in the frequency of CD4+CD8^+^ T cells in the spleen (*P* = 0.0662) and moderate increase in the frequency of CD4-CD8- T cells in lung tissue in SIV-infected, ART-treated macaques compared to SIV-naive macaques (*P* = 0.0312) (Fig. S3).

**FIG 9:**
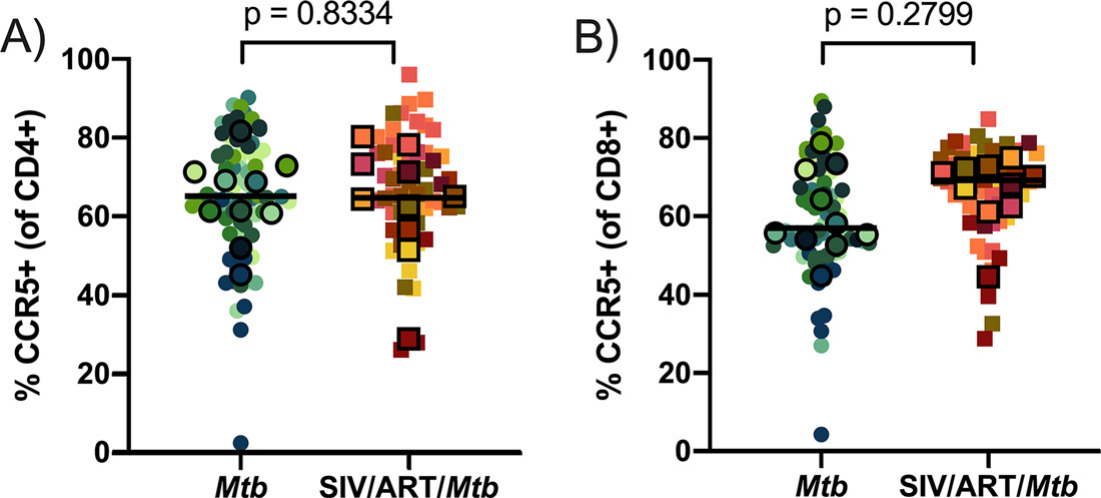
CCR5 frequency of CD4^+^ and CD8^+^ T cells isolated from granulomas. Frequencies of CCR5+ CD4^+^ and CD8^+^ T cells in granulomas. Outlined symbols indicate median per animal and unlined symbols indicate individual samples. Bars indicate group medians. (A) CCR5+ CD4^+^ T cells. An unpaired t test was performed of group medians to determine significance. (B) CCR5+ CD8^+^ T cells. A Mann-Whitney U test of group medians was performed to determine significance. *P* values are shown.

**FIG 10:**
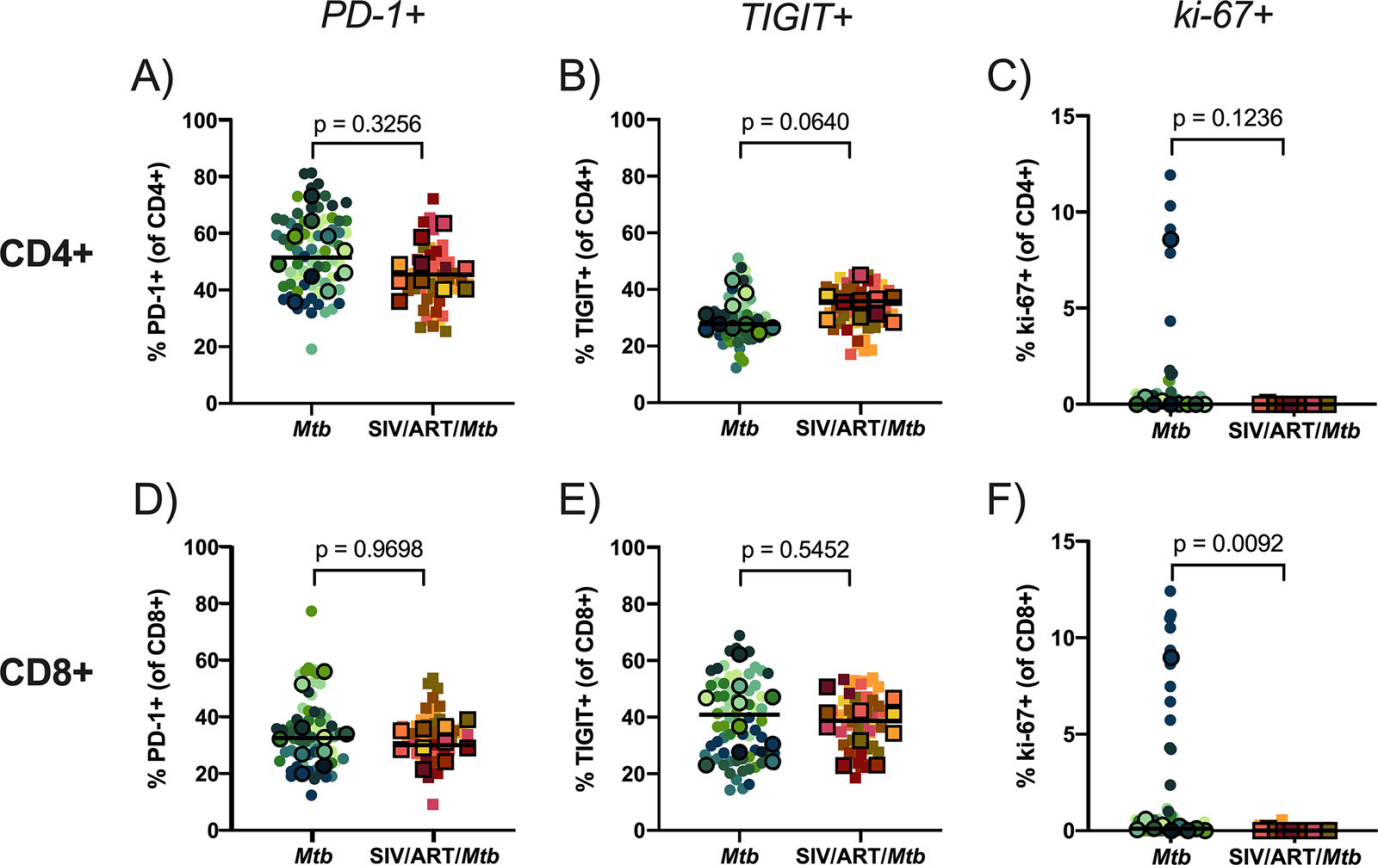
PD-1, TIGIT, and ki-67 frequency of CD4^+^ and CD8^+^ T cells isolated from granulomas. Frequencies of phenotype markers in granulomas relative to CD4^+^ or CD8^+^ gate. Outlined symbols indicate median per animal and unlined symbols indicate individual samples. Bars indicate group medians. Mann-Whitney U tests of group medians were performed to determine significance. *P* values are shown. (A) PD-1+ CD4^+^ T cells. (B) TIGIT+ CD4^+^ T cells. (C) ki-67+ CD4^+^ T cells. (D) PD-1+ CD8^+^ T cells. (E) TIGIT+ CD8^+^ T cells. (F) ki-67+ CD8^+^ T cells.

Lastly, we investigated the frequency of CCR5 and several other phenotypic markers (PD-1, TIGIT, and ki-67) on CD4^+^ and CD8^+^ T cells isolated from lung granulomas (Fig. 9 and 10). Unlike the airways, CCR5+ CD4^+^ and CD8^+^ T cells did not significantly differ between SIV-infected, ART-treated macaques and macaques infected with Mtb alone (Fig. 9A and B). For the other phenotypic markers (Fig. 10), there were no significant differences between the 2 groups apart from TIGIT+ CD4^+^ T cells (*P* = 0.0647) (Fig. 10B), which trended slightly higher in frequency in SIV/ART/Mtb macaques, and ki-67+ CD8^+^ T cells, which were significantly more frequent in granulomas from SIV-naive animals (*P* = 0.0092) (Fig. 10F). However, the difference in the median frequency of ki-67+ cells between the 2 groups was incredibly low (< 0.12% of CD8^+^ T cells) and warrants caution about ascribing biological significance to this result. Together, these data demonstrate that CD4^+^ and CD8^+^ T cells in granulomas from SIV-infected, ART-treated macaques are quite similar to those from macaques infected with Mtb alone.

Our findings show that, while some differences were detected between SIV-infected, ART-treated juvenile macaques that are coinfected with Mtb, compared to macaques infected with just Mtb, the overall immune response and TB progression appear to be remarkably similar.

## DISCUSSION

TB progression in HIV+ and HIV-naive children is not well understood due to challenges in diagnosis, monitoring, and limited epidemiological data (1, 61). Studies from the pre-antibiotic era have noted that infants and young children are at greater risk of developing pulmonary TB and more severe, disseminated forms of TB (e.g., miliary TB or TB meningitis) (9). This risk declines between the ages of 2 and 10 followed by an increased risk of pulmonary TB during puberty (9). HIV increases TB risk and results in more severe TB disease (2–4, 10, 11). It has yet to be thoroughly investigated how TB, both on its own and with a concurrent HIV infection, manifests in the presence of a developing immune system. Conventional and unconventional T cell compartments are in constant flux throughout childhood (13–16), highlighting the need to elucidate immune mechanisms behind TB pathogenesis where adult models may fall short. Only a handful of studies have modeled TB progression using young animals (45–47) and there are no studies that have modeled the impact of HIV coinfection on TB progression in juvenile macaques.

Here, we established a model of pediatric HIV/Mtb coinfection by infecting juvenile macaques with SIV, initiating ART 3 months later, and coinfecting with Mtb 3 months later. We compared TB progression in the SIV-infected, ART-treated juveniles with that in similarly aged animals infected with Mtb alone. We chose to implement ART in this study as ART coverage in children is improving worldwide (62) and is an increasingly likely real-world scenario. Prior to Mtb coinfection, the SIV-infected animals were aviremic, indicative of successful viral control by ART. Both groups exhibited similar progression of TB disease by PET/CT, pathology scores, Mtb dissemination and Mtb burden, with the exception of lower lung CFU in SIV-infected, ART-treated animals (Fig. 5 and 6, and Fig. S2). These results are in stark contrast with our previous study of adult macaques coinfected with SIV/Mtb in which all coinfected animals developed rapid dissemination of TB within the first 8 weeks of Mtb coinfection and extensive TB pathology (40). This difference is most likely due to the ART that was provided to the SIV-infected juveniles in this study, which is consistent with the efficacy of ART reported in both adults (63, 64) and children (3–7, 65). We cannot rule out age as a potential contributor to the differences observed in TB progression in SIV-infected adult macaques (40) and the juveniles reported here since we did not coinfect SIV-infected, ART-naive juveniles. However, clinical studies suggest that HIV-naive children are slightly more susceptible, and not more resistant, to severe forms of TB than adults (8, 9). The striking degree to which TB disease in our SIV-infected juveniles paralleled that in SIV-naive juveniles suggests strongly that ART may be responsible for constraining TB progression in SIV-infected juvenile macaques.

In humans, ART only partially restores immune function and resistance to Mtb in HIV+ subjects (63, 64). Thus, we characterized the T cell responses to Mtb in the SIV-infected, ART-treated animals relative to those in animals infected with Mtb alone. Since Mtb primarily establishes infection in the lung, we investigated T cell-mediated immunity in the airways throughout the study and in granulomas and lung tissue harvested 12 weeks after Mtb infection. We showed previously that SIV-related changes occur across T cell subsets in several lung compartments, including airways, granulomas, and lung tissue, in SIV-infected, ART-naive adult macaques coinfected with Mtb (44). Adult macaques coinfected with SIV/Mtb exhibited fewer CD4^+^ T cells in blood and more CD8^+^ T cells in airways (44). Similarly, in the SIV-infected juvenile macaques here, we observed decreased CD4^+^ T cells and increased CD8^+^ T cells in the airways prior to ART initiation. However, following ART treatment, both T cell types returned to pre-SIV levels indicating a restoration of CD4^+^ and CD8^+^ T cell levels in the airways by ART. The unconventional T cell populations, Vγ9+ γδ T cells, MAIT and NKT cells, which are widely thought to play a role in early Mtb infection (29–33), varied in frequency between animals and there was no consistent change in these populations during SIV infection, ART, or Mtb coinfection. Following Mtb infection, no striking differences were observed between the groups in either conventional T cell subsets or unconventional T cells in the airways (Fig. 2), aside from the elevated CD4+CD8^+^ T cells that is likely due to Mtb-mediated immune activation (48).

In addition to analyzing cells from the airway, we analyzed both lung tissue and individual granulomas. The latter is especially important since substantial heterogeneity can exist between granulomas, even from within the same animal (66). We did not observe differences in granuloma composition of T cell subsets, conventional or unconventional, nor were there any consistent differences in other tissues analyzed between SIV-naive and SIV/ART-treated Mtb coinfected macaques. IFNγ and TNF responses upon Mtb-specific stimulation of CD4^+^ and CD8^+^ T cells isolated from lungs were also similar between SIV-infected, ART-treated macaques coinfected with Mtb and macaques infected with Mtb alone. Both cytokines are associated with Mtb control (67, 68). Notably, we previously identified a defect in TNF production in SIV/Mtb coinfected adult macaques which may, at least in part, be responsible for the loss of control over Mtb in SIV-infected animals (44). A similar TNF defect in lymphocytes from lung tissue was not observed in the SIV-infected juvenile macaques studied here and may be attributed to the ART regimen. Mtb-specific responses in CD4^+^ and CD8^+^ T cells in blood from HIV/Mtb coinfected individuals have been shown to increase following ART, indicative of restoration of immune responses (69, 70). Consistent with other studies in human and NHP (26, 71), our data suggests that these similarities in IFNγ and TNF may be attributed to immune restoration from the ART regimen.

Chronic immune activation has been implicated in immune dysfunction associated with HIV/SIV infection (72, 73). PD-1 and TIGIT are inhibitory receptors that are critical in modulating immune activation and play an important role in Mtb control (74–77). Importantly, PD-1 and TIGIT expression can be upregulated on both exhausted, as well as activated, T cells. Previously, we found that frequencies of PD-1+ and TIGIT+ T cells are notably higher in lung tissue and granulomas from SIV-infected, ART-naive adult macaques and appear to be activated, regardless of whether they are coinfected with Mtb (44). This is consistent with the hypothesis that this immune activation phenotype is due to chronic SIV infection and is maintained throughout SIV/Mtb coinfection (44, 73). Interestingly, in this study, there was no difference between the 2 groups of juvenile macaques in PD-1 or TIGIT expression on CD4^+^ and CD8^+^ T cells from either airways or granulomas, aside from the decline in PD-1+ CD4^+^ T cells in airways following Mtb infection. Signaling pathways associated with inflammation and immune activation have been shown to decline during the first 6 months of ART in whole blood from HIV-1/Mtb coinfected individuals (78). Similarly, our data suggest that ART may have alleviated the chronic immune activation associated with uncontrolled viral replication.

We did observe transient changes in proliferation and cell trafficking in airways over the course of the SIV and Mtb infections. Proliferation of both CD4^+^ and CD8^+^ T cells spiked 4 weeks after SIV infection, which closely mirrored the spike in plasma viremia. Similar elevations in CD4^+^ and CD8^+^ T cell proliferation were previously observed in blood, lymph nodes, and gut shortly after SIV infection (49–51). This burst of T cell proliferation corresponds to elevations in T cell apoptosis (50) and likely indicates a compensatory response to restore CD4^+^ T cell levels depleted by rampant viral replication, while simultaneously boosting CD8^+^ T cells, well-known antiviral effectors cells. We observed an increase in the frequency of CCR6+CD4^+^ and CCR6+CD8^+^ T cells in both groups following Mtb infection. CCR6 is a chemokine receptor expressed on both dendritic cells and T cells (52). CCR6 expression on T cells promotes migration of Th1 and Th17 responses to sites of inflammation and it is associated with Mtb control (53). In our study, ART-treated SIV infection did not appear to affect the expression of CCR6 on CD4^+^ and CD8^+^ T cells in the airways during Mtb coinfection compared to animals infected with Mtb alone.

CCR5 is an important chemokine receptor for T cell migration during inflammation and is a co-receptor for HIV/SIV (22). Mtb-specific CD4 T cells upregulate CCR5 during latent TB infection and are preferentially depleted during HIV infection (79, 80). We observed a decline in the frequency of CCR5+ CD4^+^ T cells as well as a subtle, but significant, loss of absolute CCR5+ CD4^+^ T cells in the airways of SIV-infected, ART-treated macaques following Mtb coinfection. However, we did not observe a loss of CCR5+ CD4^+^ T cells in granulomas. CCR5 is expressed at higher frequencies on T cells in airways compared to other compartments like blood (81). It is possible that the elevated expression of CCR5 on CD4 T cells in airways combined with Mtb coinfection created a target-rich environment for SIV, thereby resulting in their selective depletion (22). Interestingly, others have shown that CCR5+ CD4^+^ T cells are rapidly depleted in granulomas from untreated Mtb/SIV coinfected macaques (82). These data suggest that ART may have prevented depletion of CCR5+ CD4^+^ T cells in granulomas of our SIV-infected macaques. CCR5 is also expressed on type 1 CD8^+^ T cells, which largely secrete IFNγ (55). SIV infection has been reported to cause significant depletion of CCR5+ CD8^+^ T cells at mucosal sites, such as jejunum (55). Others have reported CCR5 expression decline on circulating CD8^+^ T cells from HIV-infected progressors (83). We observed a significant decline in the frequency of CCR5+ CD8^+^ T cells and total CCR5+ CD8^+^ T cells in the airways after Mtb coinfection in SIV-infected, ART-treated juvenile macaques. It is possible that CCR5 was downregulated on CD8^+^ T cells during Mtb coinfection, however, the decline of absolute CCR5+ CD8^+^ T cells suggests a loss of these cells. In granulomas, on the other hand, we did not observe a significant loss of CCR5+ CD8^+^ T cells. Unlike CD4^+^ T cells, which have a clear mechanism of depletion via direct viral infection, the mechanism behind this tissue-dependent loss of CD8^+^ T cells during SIV infection is not well understood and warrants further investigation. Nevertheless, TB outcome did not differ between the 2 groups, indicating that the loss of CCR5+ T cells in the airways did not exert a substantial effect on TB resistance.

In summary, we found that SIV-infected, ART-treated juvenile macaques control TB similarly to juvenile macaques infected with Mtb alone. Further, immune responses to Mtb do not appear to be substantially impaired in SIV-infected juvenile macaques on ART. One limitation of our study was that we did not have an ART-naive group of SIV-infected juveniles with which to directly compare the effect of ART on TB pathogenesis in SIV-infected juvenile macaques. The median age of our SIV-infected animals also was slightly older than our SIV-naive animals due to issues with animal availability. Despite this minor age difference, the disease outcome was still similar in both groups. There is ample evidence in humans (69, 70, 78) that supports our hypothesis that ART may have restored anti-Mtb immunity and control of TB in SIV-infected juvenile macaques. ART previously was shown to reduce the risk of SIV-induced reactivation of latent TB infection (LTBI) in adult macaques (71), and ART may be an important tool to reduce TB reactivation in HIV+ people with LTBI (84). Our study suggests that early implementation of ART can restore the rate of TB progression in macaques already infected with SIV to that of SIV-naive macaques, and the first time ART has been studied in a pediatric coinfection model. Whether later ART initiation would result in a similar host responses and rate of TB progression warrants further investigation. Although, early ART has been noted to have beneficial effects on CD8 T cells in HIV-infected children (85). Our data indicate that early ART initiation provides not only viral control but may also reduce TB severity and mortality in children living with HIV. Indeed, ART is highly beneficial in reducing mortality in children infected with HIV (86). Unfortunately, ART coverage worldwide in children still lags behind adults (62) and this study further justifies increasing ART coverage in this vulnerable population.

## MATERIALS AND METHODS

### Animal studies.

Juvenile (~ 1 to 2 years of age, equivalent to children 4 to 8 years old) Mauritian cynomolgus macaques (Macaca fascicularis) were obtained from Bioculture US (Table S1). MHC haplotype was determined by MiSeq sequencing and animals with the presence of at least 1 copy of the M1 MHC haplotype were selected for this study (87).

Animal protocols and procedures were approved by the University of Pittsburgh Institutional Animal Care and Use Committee (IACUC) which adheres to guidelines established in the Animal Welfare Act and the Guide for the Care and Use of Laboratory Animals, as well as the Weatherall Report (8th Edition). The University is fully accredited by AAALAC (accreditation number 000496), and its OLAW animal welfare assurance number is D16-00118. The IACUC reviewed and approved the study protocols 19014337 and 22010433, under Assurance Number A3187-01.

Animal welfare was monitored as described previously (44). In brief, all animals were checked at least twice daily to assess appetite, attitude, activity level, and hydration status, etc. Following Mtb infection, the animals were monitored closely for clinical signs of TB (e.g., weight loss, tachypnea, dyspnea, or coughing). Physical exams, including weights, were performed on a regular basis. Animals were sedated for all veterinary procedures (e.g., blood draws) using ketamine or other approved drugs. Regular PET/CT imaging was conducted and has proven to be very useful for monitoring TB progression. Our veterinary technicians monitored animals especially closely for any signs of pain or distress. If any were noted, appropriate supportive care (e.g., dietary supplementation and rehydration) and treatments (analgesics) were given. Any animal considered to have advanced disease or intractable pain from any cause, was deemed to have reached the humane endpoint, sedated with ketamine and humanely euthanized using sodium pentobarbital.

### SIV and Mtb infections of macaques.

One group of juvenile macaques was infected intravenously with SIVmac239M (10,000 IU) (Fig. 1A). SIVmac239 is a molecularly barcoded virus stock generated from clonal SIVmac239 (88). A daily ART regimen of dolutegravir (DTG; 2.5 mg/mL, *s.c.*), tenofovir disoproxil fumarate (TDF; 5.1 mg/mL, *s.c.*), and emtricitabine (FTC; 40 mg/mL, *s.c.*) (89) was initiated 3 months after SIV infection and continued for the remainder of the study. DTG was kindly provided by ViiV Healthcare and TDF and FTC were kindly provided by Gilead.

For Mtb infection, SIV-naive (*n* = 10) and SIV-infected (*n* = 10) juvenile animals were infected with a low dose (5 to 11 CFU) of Mtb Erdman via bronchoscopic instillation, as described previously (56), and followed for 12 weeks post Mtb infection (Fig. 1A).

### Plasma viral load analysis.

For animals infected with SIV, plasma viral load was quantified by quantitative reverse transcription-PCR (qPCR) as previously described (90). In brief, plasma was isolated from whole blood by Ficoll-based density centrifugation. Viral RNA (vRNA) was isolated from cryopreserved plasma samples using the Maxwell Viral Total Nucleic Acid Purification kit (Promega). Then, vRNA was reverse transcribed using the TaqMan Fast Virus 1-Step qRT-PCR kit (Invitrogen) and quantified on a LightCycler 480 instrument (Roche). Primers and probes used for qPCR are described in (91). qPCR was performed in triplicate for each sample. The limit of detection was established by performing serial dilutions of a known standard.

### Clinical and microbiological monitoring.

All animals were assessed twice daily for general health and monitored closely for clinical signs of TB (coughing, weight loss, tachypnea, and dyspnea etc.) following Mtb infection. Monthly gastric aspirates (GA) and bronchoalveolar lavage (BAL) samples were tested for Mtb growth. GA and BAL samples with culturable Mtb (+) or that were sterile (-) are indicated in Table S1. Blood was drawn at regular intervals to measure erythrocyte sedimentation rate (ESR) and to provide peripheral blood mononuclear cells (PBMC) and plasma. No animals exhibited an elevated ESR over the course of the study, indicated in Table S1 with a negative sign (-).

### PET/CT imaging and analysis.

Radiolabeled 2-deoxy-2-(^18^F)fluoro-d-glucose (FDG) PET/CT was performed just prior to Mtb infection and then monthly after Mtb infection. Imaging was performed using a MultiScan LFER-150 PET/CT scanner (Mediso Medical Imaging Systems) housed within our BSL3 facility as previously described (92, 93). Co-registered PET/CT images were analyzed using OsiriX MD software (version 12.5.2, Pixmeo) to enumerate granulomas and to calculate the total FDG avidity of the lungs, exclusive of lymph nodes, which is a quantitative measure of total inflammation in the lungs (92, 94). Thoracic lymphadenopathy and extrapulmonary dissemination of Mtb to the spleen and/or liver were also assessed qualitatively on these scans.

### Necropsy.

Necropsies were performed as previously described (40, 44) 12 weeks after Mtb infection. One SIV-infected, ART-treated animal (35319) met humane endpoint criteria and necropsied 6 weeks after Mtb coinfection (Table S1). A final FDG PET/CT scan was performed no more than 3 days prior to necropsy to document disease progression and to guide collection of individual granulomas (56). Animals were heavily sedated with ketamine, maximally bled, and humanely euthanized using sodium pentobarbital (Beuthanasia, Schering-Plough). Granulomas matched to the final PET/CT images were harvested along with other TB pathologies (e.g., consolidations and pneumonia), thoracic and extrathoracic lymph nodes, lung tissue, as well as portions of liver and spleen. Quantitative gross pathology scores were calculated and reflect overall TB disease burden for each animal (56). Briefly, pathology scores are separated in 3 components: lung, thoracic lymph nodes (LN), and extrapulmonary (EP). Lung pathology scores are based on the number and size of granulomas, and whether there was advanced pathology (e.g., consolidations and pneumonia). Thoracic LN pathology was scored based on granuloma size and number, as well as the number of LN involved. EP disease score reflects the number of granulomas and the number of EP sites involved. Tissue samples were divided, and a portion was fixed in 10% neutral buffered formalin (NBF) for histopathology; the remainder was homogenized to a single-cell suspension as described previously (56). Serial dilutions of these homogenates were plated onto 7H11 agar, incubated at 37°C, 5% CO_2_ for 3 weeks, and colonies were enumerated. Samples yielding colonies were considered CFU+. Bacterial load in lungs, thoracic lymph nodes, liver, and spleen, as well as total thoracic CFU, were calculated as described previously (48). NBF-fixed tissue was embedded in paraffin, sectioned, and stained with hematoxylin and eosin for histopathologic examination.

### Flow cytometry of BAL and tissue.

In general, cells collected from BAL and necropsy were stained following a similar protocol. For BAL, we instill four (4) 10-mL saline washes of the airway, recovering as much fluid as possible between each wash. Cells were pelleted and counted using a hemocytometer, with yields reported as total cell number. Cells were then aliquoted at 1×10^6^ cells/well in a 96-well plate and stained immediately to ensure proper assessment of conventional and unconventional T cell subsets. For stimulation assays assessing cytokine production, cells from lung lobes, lymph nodes, and spleen were divided into wells containing either media, Mtb H37Rv whole cell lysate (20 μg/mL; BEI Resources) or M. smegmatis (MOI 5:1) and stimulated for 14 h total at 37°C. In brief, stimulators were added and incubated for 2 h, then brefeldin A (1 μg/mL) was added for the remainder of the stimulation time. Cells were reconstituted in 500 nM dasatinib in ELISpot media (RPMI 1640 + 10% human albumin + 1% glycine + 1% HEPES buffer) to improve tetramer staining. MR1 (5-OPRU; PE) and CD1d (PBS-57; APC) tetramers were added to wells and incubated for 30 min at room temperature (NIH Tetramer Core Facility). Antibody for Vα7.2 (Table S2) was added, incubated for an additional 30 min at room temperature, and washed twice with PBS containing dasatinib. Cells were stained with a viability dye (Table S2) for 10 min at room temperature and washed with FACS containing dasatinib. Cells were stained with surface antibody cocktail (Table S2) for 20 min at 4°C, were fixed in 1% PFA, and permeabilized with BD Cytofix/Cytoperm (BD; Cat No. 554714) for 10 min at room temperature. Cells were stained intracellularly for 20 min at room temperature, washed, and analyzed immediately.

Flow cytometry of BAL and tissue samples was performed using a Cytek Aurora (BD). FCS files were analyzed using FlowJo software for Macintosh (version 10.1). Gating strategies for BAL and tissue data are shown in Fig. S4 and 5, respectively. For markers without a clearly separated positive population (e.g., ki-67, TIGIT, CCR5, CCR6, and CXCR3), gates were set along the outer edge of the negative population visible in the pseudocolor plot. For cytokines, positive gates were set according to unstimulated samples. For most samples, we acquired 50,000 events in the lymphocyte gate. However, when this was not possible (i.e., for some small granulomas), we applied a cutoff threshold of CD3 events > 100 to ensure precise and accurate measurements of phenotype and cytokine markers, as previously described (48, 66, 95). Samples below that threshold were excluded from further analysis. Absolute CCR5+ CD4^+^ and CD8^+^ T cell counts were calculated by multiplying the frequency of CCR5+ or CCR5- CD4^+^ and CD8^+^ T cells from the live leukocyte gate with the absolute number of BAL cells recovered. Cytokine production was background-adjusted by subtracting cytokine frequencies of unstimulated conditions (media only) from cytokine frequencies of stimulated conditions. Negative values were corrected by establishing a threshold based on the distribution of the negative values. Values below this threshold (absolute value of the 75^th^-percentile of negative values) were then adjusted to zero (44, 96).

### Sequencing of barcode identifiers in Mtb.

Mtb genomic DNA was isolated as previously described (58) and Mtb barcodes were sequenced as previously described (97). Only CFU+ samples were included in Mtb barcode analyses. Briefly, genomic DNA was isolated, and samples were quantified and diluted to 10 ng/μL. Samples were then amplified twice using 2x Q5 Master Mix (New England BioLabs) and 2 unique primer sets, 1 to add a molecular counter to distinguish unique input templates, and 1 to add the Illumina TruSeq adapter sequences, were used. Primer sequences can be found in Table S3. Samples were then sequenced using a 2 × 150 MiSeq cartridge with v2 chemistry at a concentration of 4 pM and a 20% PhiX spike. A computational pipeline courtesy of Dr. Michael Chase and the Fortune lab was used to determine the number and proportion of unique barcode sequences in each sample from FASTQ files (97). Only barcodes present at a frequency of 1% or greater were included. All figures were generated using Prism 9 and Adobe Illustrator 2019.

### Statistics.

For comparing longitudinal BAL data, linear mixed models with subject as a random variable were used to test treatment groups over time (Table S4). Fixed effect tests were used to assess whether there were differences among treatment groups or among time points. All time points were then compared with Tukey HSD (honestly significant difference) test using the Tukey-Kramer multiple comparison adjustment. Linear models were run in JMP Pro (v.14.3.0). A two-way repeated measures ANOVA was used to test if there were differences in the number of barcodes between infection group (Mtb versus SIV/ART/Mtb) and tissue type (granulomas versus thoracic lymph nodes) and Bonferroni’s multiple comparison adjusted *P* values were reported (Fig. S2B).

For all other data, the Shapiro-Wilk normality test was used to check for normal distribution of data. Unpaired normally distributed data were analyzed using t tests, while unpaired non-normally distributed data were analyzed with the Mann-Whitney U test. Statistical tests were performed in Prism (version 8.2.1; GraphPad). All tests were two-sided, and statistical significance was designated at a *P* value of < 0.05. *P* values between 0.05 and 0.10 were considered trending.

### Data availability.

All Mtb barcode sequences are available on the Sequence Read Archive (SRA) under accession number PRJNA900591.
