# Supplementary figures and images for: Host Immunity to Mycobacterium tuberculosis Infection Is Similar in Simian Immunodeficiency Virus (SIV)-Infected, Antiretroviral Therapy-Treated and SIV-Naïve Juvenile Macaques
Source: Infect Immun. 2023 Apr 11;91(5):e00558-22. doi: 10.1128/iai.00558-22 (PMC10187125; doi:10.1128/iai.00558-22)

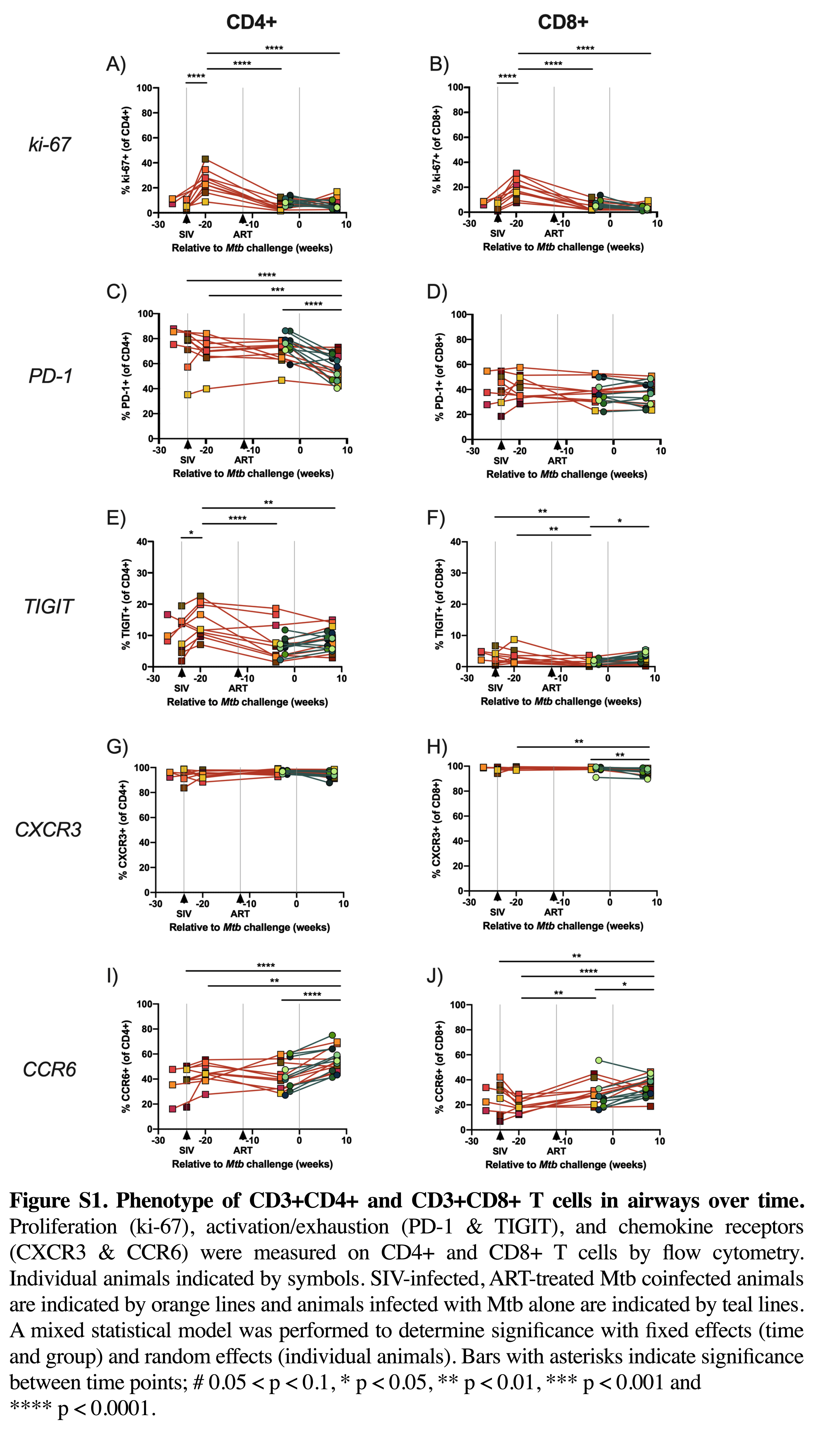

Supplement: Supplemental file 5 — Fig. S1 to S5. Download iai.00558-22-s0005.tif, TIF file, 13.9 MB [file iai.00558-22-s0005.tif]
